# Long‐Term Local Control and Mortality After Transpupillary Thermotherapy of Small Uveal Melanomas

**DOI:** 10.1111/ceo.70126

**Published:** 2026-05-08

**Authors:** Maria Fili, Malin Ermedahl Conradi, Maya Klaff Dahl, Shiva Sabazade, Manuel Casselholm De Salles, Gustav Stålhammar

**Affiliations:** ^1^ Department of Clinical Neuroscience, Division of Eye and Vision Karolinska Institutet Stockholm Sweden; ^2^ Ocular Oncology Service, St. Erik Eye Hospital Stockholm Sweden; ^3^ St. Erik Ophthalmic Pathology Laboratory St. Erik Eye Hospital Stockholm Sweden

**Keywords:** choroidal melanoma, local recurrence, mortality, thermotherapy, TTT, uveal melanoma

## Abstract

**Background:**

Transpupillary thermotherapy (TTT) is used for selected small choroidal melanocytic tumours, either as primary treatment or as an adjunct to plaque brachytherapy. We compared long‐term local recurrence and mortality outcomes after primary TTT alone, plaque brachytherapy combined with TTT (brachy+TTT), and plaque brachytherapy alone (brachy alone).

**Methods:**

This retrospective cohort study included 850 patients with choroidal melanocytic tumours treated with eye‐preserving modalities at St. Erik Eye Hospital, Stockholm, Sweden, 2008–2020. Primary treatment was TTT alone (*n* = 48), brachy+TTT (*n* = 105), or brachy alone (*n* = 697). Outcomes included local recurrence, local recurrence with extraocular extension (EXE), and mortality.

**Results:**

At 15 years, Kaplan–Meier local recurrence‐free survival was 8% after TTT alone, 34% after brachy+TTT, and 86% after brachy alone (log‐rank test for trend, *p* < 0.001). Disease‐specific survival at 15 years was 46%, 85%, and 90%, respectively (*p* = 0.03). In multivariable Fine–Gray regression adjusting for American Joint Committee on Cancer stage, TTT alone was associated with a higher subdistribution hazard of local recurrence (sHR, 6.7; *p* < 0.001) and melanoma‐related death (sHR, 3.9; *p* < 0.001). Among patients treated for local recurrence, subsequent recurrences occurred more frequently after TTT than after treatments involving brachytherapy. The risk of EXE increased with the number of TTT sessions.

**Conclusions:**

In this single‐centre retrospective cohort, TTT was associated with higher rates of local recurrence and melanoma‐related mortality compared with plaque brachytherapy. Future studies should assess whether similar results are observed in external long‐term cohorts; such findings would inform whether the role of TTT in the management of choroidal melanocytic tumours warrants reconsideration.

## 1 Introduction

Most patients diagnosed with choroidal melanoma are treated with eye‐preserving modalities such as plaque brachytherapy, proton beam irradiation, or Gamma Knife stereotactic radiosurgery [1, 2]. In 1995, transpupillary thermotherapy (TTT) using an infrared 810 nm diode laser was introduced as a conservative, eye‐preserving treatment for pigmented tumours, allowing precise, lens‐guided delivery to the tumour surface [3]. TTT delivers heat using a relatively large laser spot of 2–3 mm, low irradiance, and long exposure times, producing sub‐photocoagulation temperatures of roughly 45°C–65°C within the tumour [2]. By treating until a subtle grey‐white discoloration of the tumour surface appears, the surgeon receives real‐time visual feedback on treatment intensity and coverage, permitting careful control of the treatment area [3]. TTT can be performed as an outpatient procedure under local anaesthesia, such as retro‐ or peribulbar block or even topical drops, whereas we have elected general anaesthesia in many cases to allow for control of eye movements and to reduce discomfort [2, 3, 4].

At our institution, TTT has rarely been used as the sole treatment for unequivocal melanomas. Exceptions have included very small tumours and atypical choroidal nevi with concerning features such as orange pigment, subretinal fluid, or documented growth, as well as selected cases of small tumours in elderly or frail patients in whom general anaesthesia was unsuitable. More commonly, TTT has been used as an adjunct to plaque brachytherapy, particularly for central tumour margins near sensitive structures such as the fovea and optic disc, where TTT can be applied with precision while minimizing the risk of radiation‐induced damage [5, 6]. TTT has also frequently been used to treat small marginal recurrences after brachytherapy.

Previous reports have described relatively high rates of local tumour recurrence after TTT alone. Aaberg and colleagues reported a 10‐year Kaplan–Meier recurrence of 33% in 135 patients (mean thickness 2.2 mm, range 0.25–6.6 mm; mean diameter 8.5 mm, range 2.8–13.8 mm) [4]. Mashayekhi and colleagues reported 42% in their earlier period (mean thickness 2.7 mm, range 1.0–5.4 mm), dropping to 15% in a later period when its use was restricted to thinner (mean thickness 2.2 mm, range 1.0–4.0 mm), more peripheral tumours with fewer risk factors for growth [7]. Parrozzani and colleagues reported complications in their cohort of 77 treated eyes, including macular pucker (11 eyes), retinal vein occlusion (6 eyes), macular oedema (3 eyes), and neovascular glaucoma (3 eyes) [8]. Spire and colleagues reported a 2‐year metastasis‐free survival of only 44% in 18 patients with tumours of mean largest basal diameter 6.7 mm and thickness 3.2 mm treated with primary TTT, a figure that appears implausibly low [9]. Several case reports and small series have also described recurrences with extraocular extension (EXE) containing viable tumour following TTT [9, 10, 11, 12]. However, the evidence base remains limited by very small cohorts, short follow‐up, or the absence of comparison groups treated with other modalities, making it difficult to judge the relative performance of TTT [4, 7, 8, 9, 13, 14].

In this study, we examine the incidence of local recurrence and local recurrence with EXE after primary treatment of choroidal melanoma with TTT and compare these outcomes with those after plaque brachytherapy combined with TTT (brachy+TTT) or brachytherapy alone (brachy alone). We also evaluate whether TTT is effective when used to treat local recurrences. Because local recurrence has been associated with metastatic death, we further assess the incidence of metastatic death after each treatment modality [15, 16]. Both matched and unmatched cohorts are analysed, using the longest available follow‐up for TTT‐treated patients published to date. The findings have led us to discontinue the use of TTT for choroidal melanoma in our clinical practice altogether.

## 2 Methods

### 2.1 Hypotheses

In this study, we aim to test four hypotheses: whether primary treatment with TTT alone for choroidal melanocytic tumours is associated with (i) a higher rate of local recurrence, (ii) a higher rate of local recurrence with EXE, (iii) a higher rate of metastatic death, and (iv) whether use of TTT to treat a local recurrence is associated with a higher rate of subsequent local recurrence. Each comparison is with plaque brachytherapy, either as primary treatment (i–iii) or as recurrence treatment (iv).

### 2.2 Study Design and Data Sources

This retrospective study is based on a chart and medical record review of all patients treated with primary TTT at St. Erik Eye Hospital, Stockholm, Sweden, between January 1st, 2008, and December 31st, 2020. For comparison, we included patients treated with brachy+TTT as well as those treated with brachy alone during the same period. These patient groups were used for both unmatched analyses and matched analyses, in which patients were matched based on factors relevant to outcomes after eye‐preserving treatment of choroidal melanomas.

### 2.3 Inclusion and Exclusion Criteria

Inclusion criteria were as follows:

1. A diagnosis of choroidal melanoma or atypical choroidal nevus established by ocular oncologists after multimodal examination (e.g., ultrasonography, optical coherence tomography, fundus photography, and, when indicated, biopsy).
2. Primary treatment with an eye‐preserving modality, which at our institution consisted of TTT alone, brachy+TTT, or brachy alone.
3. Diagnosis and primary treatment between January 1st, 2008, and December 31st, 2020, as earlier records may not reliably capture complete treatment data, recurrence events, or follow‐up information.
4. Availability of sufficient clinical data to determine treatment modality, baseline tumour characteristics, and follow‐up outcomes.

A total of 977 patients met these inclusion criteria. Patients who underwent primary enucleation during the study period were held in a separate institutional register and were not part of this cohort, as they did not meet criterion 2. Patient‐driven choice of enucleation over a recommended eye‐preserving modality is extremely rare at our institution. No patients meeting criteria 1–3 were excluded because of incomplete data (criterion 4). We acknowledge that it may be debated what exactly constitutes an atypical choroidal nevus and how it should be separated from melanoma, and we avoid using the term ourselves [17]. It is included here because that was the term used by our ocular oncology colleagues for specific cases at the time. We use the term “choroidal melanocytic tumour” for all melanocytic neoplasms, regardless of the degree of malignancy.

Exclusion criteria were:

1. Metastatic disease at the time of diagnosis (*n* = 7).
2. Tumours located entirely anterior to the equator (*n* = 118) are not candidates for TTT and follow different treatment pathways.
3. Previous melanoma treatment in the study eye, including cases listed as having received primary treatment during 2008–2020 but found on review to have undergone earlier interventions (*n* = 2).

After applying these exclusions, 850 patients remained in the study cohort. Of these, 48 had undergone primary treatment with TTT alone, 105 had received brachy+TTT, and 697 had been treated with brachy alone. This study was approved by the Swedish Ethical Review Authority (reference 2025–06890‐01) and adhered to the tenets of the declaration of Helsinki. The Strengthening the reporting of observational studies in epidemiology (STROBE) checklist for cohort studies was used.

### 2.4 Transpupillary Thermotherapy and Follow‐Up Practices

At our institution, TTT for choroidal melanocytic tumours is performed using an 810 nm infrared diode laser (Iridex Corporation, Mountain View, California, USA), coupled either to a Carl Zeiss operating microscope (Carl Zeiss, Jena, Germany) in the operating room, or to a Haag‐Streit slit‐lamp biomicroscope (Haag‐Streit, Basel, Switzerland) in the outpatient clinic via an Iridex EasyView slit‐lamp adapter (Haag‐Streit style). After pharmacologic dilation, laser energy is delivered in confluent, overlapping spots with a beam diameter typically between 2.0 and 3.0 mm, covering the entire tumour and a narrow margin (~0.5–1.0 mm) of surrounding clinically normal fundus. Delivery is through one of three contact lenses, selected according to tumour location: an Ocular Reichel‐Mainster 1X Retina Laser Lens (Ocular Instruments, Bellevue, Washington, USA) as the standard lens for posterior choroidal melanomas, an Ocular OMRA‐S Mainster Focal/Grid laser lens (Ocular Instruments) for focal macular treatment, or a Goldmann three‐mirror contact lens for peripheral lesions or peripheral portions of lesions. The Reichel‐Mainster 1X and OMRA‐S lenses both provide a laser spot magnification of 1.05×. Each spot is applied for approximately 1 min, and the power is titrated to achieve a subtle grey–white discoloration of the tumour surface while avoiding frank photocoagulation, providing real‐time feedback on treatment intensity and coverage. Most patients undergo primary TTT under general anaesthesia (41 of 48 in this study) to allow stable fixation and reduce discomfort, although selected patients (7 of 48) are treated under topical anaesthesia with 0.5% tetracaine hydrochloride at the slit lamp. Typical indications for primary TTT at our centre have included small, predominantly posterior choroidal melanomas with an ultrasound thickness < 3 mm, often located in the macular or peripapillary region, as well as atypical choroidal nevi followed over time that have developed risk factors such as orange pigment, subretinal fluid, symptoms, or documented growth. Posterior location is preferred and by far the most common because such tumours are directly accessible through the pupil, but TTT has also been delivered to tumours extending anterior to the equator when they could be reached with the laser beam, in rare cases using a Goldmann three‐mirror contact lens. The term ‘small’ is used throughout this manuscript without a fixed numerical threshold, reflecting both the inconsistent use of the term in the literature and the case‐by‐case nature of treatment selection at our centre. Where size criteria from specific published series are cited, those criteria are stated; the size distribution of our own primary TTT cohort is shown in Supplementary Table S1 and Table 1. In some cases, TTT has been used as the sole treatment modality; in others, it has been combined with plaque brachytherapy, with TTT applied primarily to posterior tumour margins near the optic disc and fovea to allow more spatially confined treatment in regions where brachytherapy offers limited precision [5, 18]. When TTT was combined with plaque brachytherapy for tumours close to the optic disc or fovea, the plaque was typically placed eccentrically, with the posterior plaque margin approximately aligned with the posterior tumour margin, that is, a safety margin of approximately 0 mm on the posterior side. Near the fovea, this was done to limit radiation to the central retina. Near the optic disc, the eccentric placement was often a matter of necessity rather than choice, because the optic nerve sheath posterior to the globe is wider than the optic disc itself, so for a tumour margin near the optic disc, a posterior safety margin of 2 mm would place the plaque edge onto the optic nerve. TTT was applied to the tumour margin closest to the critical structure with the intention of compensating for the reduced brachytherapy coverage. It was rare that the plaque was positioned such that more than 2 mm of the posterior tumour margin was left outside the plaque footprint. For tumours not adjacent to these structures, plaque placement followed the institution's standard practice of a 2 mm safety margin on all sides, as described previously [15]. Because TTT was sometimes selected as primary treatment for lesions suspected to represent atypical choroidal nevi rather than unequivocal melanomas, this diagnostic uncertainty should, if anything, have biased survival in a favourable direction for TTT, as such lesions would be expected to include a proportion of tumours with inherently low malignant potential. Treatment settings and characteristics for the patients included in this study are described in the Results section. All patients, regardless of treatment modality, were scheduled for standardized ophthalmic follow‐up at 1, 3, 6, and 12 months after treatment, and annually or semi‐annually thereafter if local tumour control had been achieved, as described previously [19]. Semi‐annual surveillance for liver metastases using ultrasonography or computed tomography was performed for 5 years after diagnosis, and thereafter only when prompted by symptoms, palpable masses, deteriorating general health, jaundice, weight loss, or other clinical signs.

**TABLE 1:** Baseline characteristics of matched cohorts.

	TTT only, *n* = 48	Brachy + TTT, *n* = 96	Brachy only, *n* = 96
Sex, *n* (%)			
Female	20 (42)	47 (49)	54 (56)
Male	28 (58)	49 (51)	42 (44)
Age at treatment, mean (SD)	61.1 (12.7)	63.5 (12.9)	61.4 (12.3)
BCVA, mean logMAR (SD)	0.16 (0.24)	0.25 (0.26)	0.20 (0.26)
Tumour LBD, mean mm (median, range, SD)	6.7 (6.5, 2.0–14.0, 2.6)	7.8 (8.0, 2.0–14.0, 2.7)	7.6 (8.0, 3.0–14.0, 2.4)
Tumour thickness, mean mm (median, range, SD)	1.8 (1.6, 0.5–4.3, 0.8)	2.9 (3.0, 0.3–6.2, 1.1)	3.2 (3.0, 1.0–7.1, 1.3)
Tumour observation by ocular oncologist before treatment, *n* (%)^a^	32 (67)	64 (67)	58 (60)
Of these, with “atypical choroidal nevus” diagnosis at time of treatment, *n* (%)	4 (8)	2 (2)	3 (3)
Tumour distance to OD, mean mm (median, range, SD)^b^	2.1 (1.0, 0.0–7.0, 2.2)	1.4 (1.0, 0.0–6.0, 1.5)	2.5 (2.0, 0.0–8.0, 2.2)
Tumour touching the optic disc, *n* (%)	16 (33)	21 (22)	18 (19)
Tumour distance to foveola, mean mm (median, range, SD)	1.0 (1.0, 0.0–5.0, 1.4)	1.5 (0.5, 0.0–6.0, 1.9)	2.5 (2.0, 0.0–10.0, 2.5)
AJCC T‐category and Stage, *n* (%)			
T1a, Stage I	48 (100)	86 (90)	89 (93)
T2a, Stage IIA	0 (0)	10 (10)	7 (7)
Median follow‐up, years (IQR, min‐max)	8.5 (5.8–10.6, 0.9–17.8)	8.2 (5.8–11.7, 1.1–14.0)	6.2 (4.7–9.1, 1.5–14.8)

*Note:* Tumour and patient characteristics refer to the time of primary treatment.

Abbreviations: AJCC, American Joint Committee on Cancer; BCVA, best‐corrected visual acuity; Brachy, plaque brachytherapy; IQR, interquartile range; LBD, largest basal tumour diameter; OD, optic disc; SD, standard deviation; TTT, transpupillary thermotherapy.

^a^ Examined at least once at a prior visit for the same lesion.

^b^ Distance between posterior tumour margin and optic disc.

When a local recurrence was suspected on slit‐lamp examination, B‐scan ultrasonography, fundus imaging, or optical coherence tomography, the choice of treatment was typically discussed within the ocular oncology team. Treatment depended on the type and size of the recurrence, the primary treatment received, and the clinical condition of the eye. Marginal recurrences were typically treated with TTT, particularly when located close to the optic disc or fovea; this could be repeated on subsequent occasions if further marginal recurrences developed. Small central or diffuse recurrences could also be managed with TTT when judged amenable. Larger recurrences, typically those with a diameter > 5 mm, or with multifocal or irregular growth, or a thickness > 2 mm, were treated with plaque brachytherapy. In patients whose primary treatment had been TTT alone, this was the first brachytherapy session; in patients whose primary treatment had already included brachytherapy, a second plaque could be placed for a local recurrence, but if a subsequent recurrence developed, enucleation was preferred over a third plaque. Recurrences with extraocular extension were treated with enucleation. Enucleation was also performed for recurrences considered too large or too advanced for further eye‐preserving treatment, for recurrences associated with bleeding, neovascularization, or intraocular inflammation, and for eyes that had lost all useful vision or become painful.

### 2.5 Plaque Brachytherapy

The prescribed dose at our centre during the 2008–2020 study period was 100 Gy to the tumour apex for ruthenium‐106 plaques (CCA, CCB, CCX, CCZ, COB, CIA, and CIB types, Eckert & Ziegler BEBIG, Berlin, Germany), and 80 Gy to the tumour apex for iodine‐125 plaques (custom‐made in 18‐carat gold alloy to replicate the ruthenium plaque geometries, with iodine seeds from Eckert & Ziegler BEBIG), as described previously [19, 20, 21]. In the matched cohort, 84 of 96 primary plaques (88%) in the brachy+TTT group and 93 of 96 (97%) in the brachy‐alone group were CCB (20.2 mm); CCA plaques were not used for primary treatment in either group. No minimum scleral dose was applied. The prescribed apical dose was the same for patients treated with brachy alone and for those treated with brachy+TTT. However, brachy+TTT was frequently used for tumours close to the optic disc or fovea, for which the plaque was placed eccentrically, either intentionally or out of necessity because the optic nerve sheath prevents more posterior placement, with the posterior edge approximately aligned with the posterior tumour margin. Because the prescription isodose does not extend fully to the plaque edge in the lateral dimension, such eccentric placements would be expected to deliver a lower minimum tumour dose at the eccentric margin than centred placements [22]. Eccentric placement was not prospectively recorded for each case, but can be estimated from tumour location, since proximity to the optic disc (posterior tumour margin < 2 mm from the disc) and proximity to the fovea (tumour edge < 2 mm from the foveola) were the two routine drivers of eccentric placement at our centre. By this proxy, in the matched cohort 50 of 96 patients (52%) in the brachy+TTT group and 47 of 96 (49%) in the brachy alone group were treated with eccentric plaque placement at primary treatment. Dose calculation was non‐image‐based, using spreadsheet‐style point‐dose calculations along the plaque central axis. For ruthenium‐106, depth‐dose distributions were taken from the manufacturer's calibration certificate and independently verified in house, as reported separately [23, 24]. For iodine‐125, dose was calculated according to the AAPM TG‐43 U1 formalism, assuming homogeneous water surroundings [25]. The plaque geometry used at our centre has been characterized in detail by Monte Carlo simulation [26].

### 2.6 Cohort Matching

Three primary treatment cohorts were defined: (i) TTT alone, (ii) brachy+TTT, and (iii) brachy alone. Patients treated with TTT alone served as the index group for matching. To obtain comparable control cohorts, we matched each TTT‐treated patient to patients from the other two treatment groups using nearest‐neighbour matching on a Mahalanobis distance. Matching was performed separately for TTT alone versus brachy+TTT and for TTT alone versus brachy alone, using a 1:2 ratio without replacement. Matching covariates were age at diagnosis, American Joint Committee on Cancer (AJCC, 8th Edition) T‐category, and tumour distance to the optic disc (in mm). The two matched sets were then combined to form the final matched cohort used in subsequent analyses. Supplementary Table S1 describes the full unmatched cohort for completeness. With one exception, all comparative analyses were performed on the matched cohort (Table 1), restricted to T1a and T2a tumours. The single exception is a multivariable Fine–Gray regression, where AJCC stage is included as a covariate to adjust for the differences in tumour extent between treatment groups.

### 2.7 Statistical Analyses

Follow‐up time was summarized as the median observed follow‐up in years, along with the interquartile range and range. Reverse Kaplan–Meier estimates of follow‐up were also examined, but the median follow‐up time was not estimable from the reverse Kaplan–Meier curve in these data because the curve did not cross the 50% probability level. For all time‐to‐event analyses of primary treatment, follow‐up time was measured in years from the date of first treatment until the event of interest or censoring at the last documented follow‐up. Kaplan–Meier estimates for disease‐specific survival and overall survival were plotted. In competing risks regression analyses, we modelled the subdistribution hazard ratio (sHR) for first local recurrence with enucleation and death from any cause as competing events, and the sHR for melanoma‐related mortality (death from metastatic uveal melanoma) with death from any other cause as the competing event. These analyses were performed in both the full (unmatched) cohort and in a matched cohort. For patients who developed a first local tumour recurrence and subsequently received additional local treatment, the sHR for additional recurrences was evaluated using Fine‐Gray competing risks regression. For the Fine–Gray competing risks regression of additional local recurrences, time zero was the date of the recurrence‐treatment surgery, and patients who underwent more than one eye‐preserving recurrence treatment contributed one episode per treatment. Follow‐up continued until another local recurrence (event of interest), secondary enucleation or death from any cause (competing events), or the last documented follow‐up (censoring). To account for within‐patient correlation, 95% confidence intervals for the subdistribution hazard ratio were obtained by a patient‐level cluster bootstrap with 2000 resamples. Proportional subdistribution hazards assumptions for the Fine–Gray competing risks regression models were evaluated by adding interactions between treatment group indicators and log(time), followed by Wald testing of the interaction terms. Proportionality was evaluated separately for each endpoint. AJCC stage was entered as an ordinal covariate (1–6 for stages I through IIIC); linearity was verified against a categorical model by likelihood ratio test. Time‐dependent Cox regressions of disease‐specific and overall survival were fitted on the matched cohort with local recurrence as a time‐varying covariate. Individual treatment trajectories and the timing of local recurrences, secondary enucleations, and deaths in the TTT cohort were visualized using a Swimmer‐style plot with time measured from the first TTT treatment. Associations between TTT exposure and EXE were evaluated using Fisher's exact test because EXE was absent in the no‐exposure groups (complete separation), precluding finite odds ratio estimation. All statistical analyses and data management were performed in R version 4.4.3 (The R Foundation for Statistical Computing, Vienna, Austria). The following R packages were used: readxl for data import; dplyr, tidyr, stringr, MatchIt, cmprsk, ggplot2, ggsci, and lubridate. All tests were two‐sided, and a significance level of *p* < 0.05 was used. No formal adjustment for multiple comparisons was applied. All outcomes and comparisons were specified a priori and addressed distinct, clinically motivated hypotheses.

## 3 Results

### 3.1 Descriptive Statistics

Of the 850 included patients, 422 (50%) were female and 428 (50%) were male. The mean ± standard deviation (SD) age at diagnosis was 66 ± 13 years. In the unmatched cohort, patients treated with brachy alone had the largest tumours (mean largest basal diameter [LBD] 11.4 mm; mean thickness 6.0 mm). Tumours treated with brachy+TTT were smaller (mean LBD 8.3 mm; mean thickness 3.0 mm), and those treated with TTT alone were the smallest (mean LBD 6.7 mm; mean thickness 1.8 mm; Supplementary Table S1).

A total of 163 patients developed at least one local recurrence after primary treatment (36/48 after TTT alone, 28/105 after brachy+TTT, and 99/697 after brachy alone). Because some patients experienced multiple recurrences, the total number of recurrence events was 225. Of these 163 patients, 125 had one recurrence, 25 had two, eight had three, three had four, one had five, and one had nine separate local recurrences.

**FIGURE 1:**
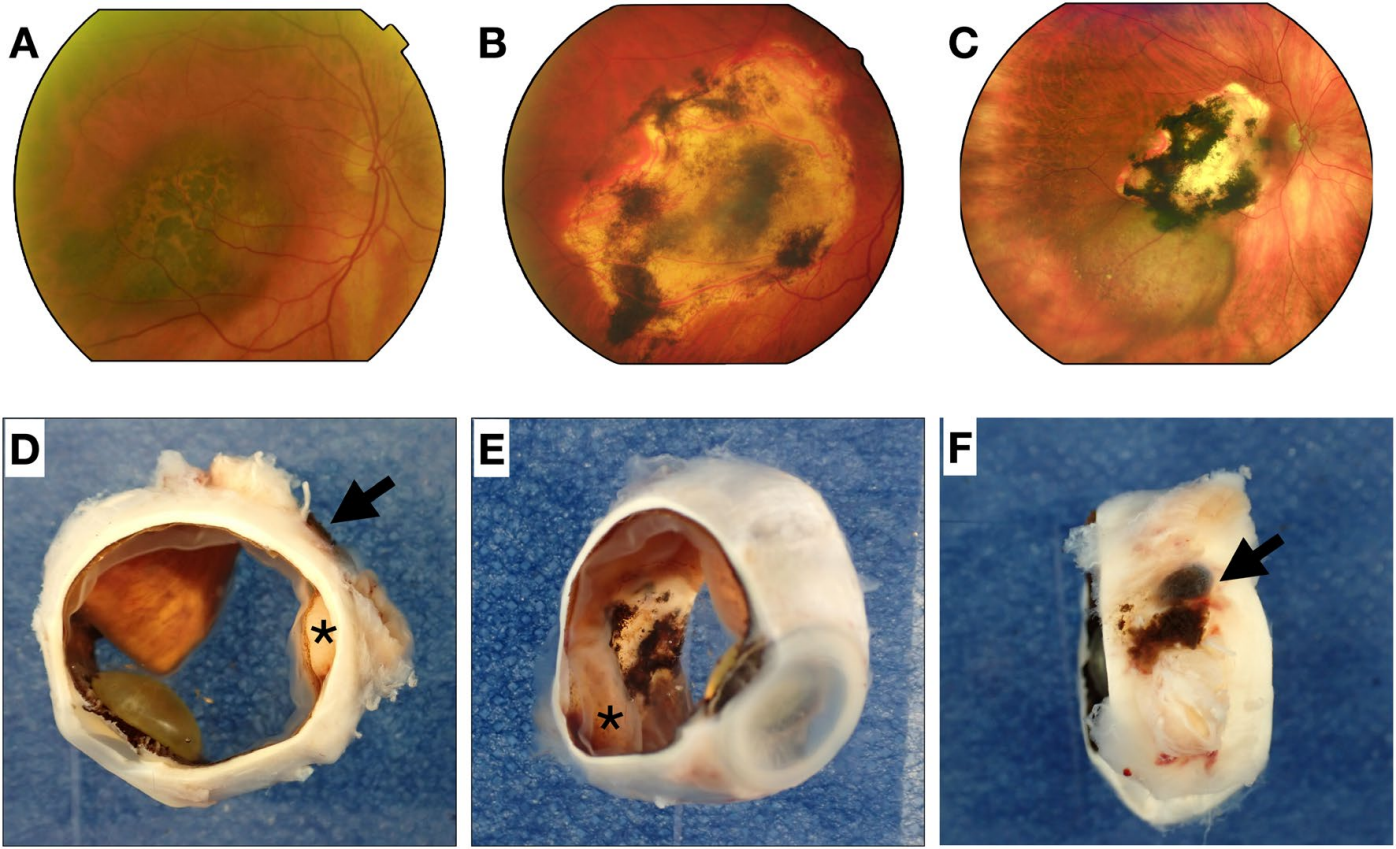
Fundus photographs and gross pathology of a choroidal melanoma treated with primary transpupillary thermotherapy (TTT). (A) A 78‐year‐old male was diagnosed with a small choroidal melanoma measuring 8 mm in largest basal diameter and < 2 mm in thickness. (B) One session of TTT was performed, after which the tumour regressed to a predominantly flat scar, with two central areas of residual pigmentation with diffuse margins. This case predates the routine use of optical coherence tomography at our centre, and OCT may have shown thin residual tumour at these sites. The later finding of extraocular extension on histopathology suggests that recurrent tumour may have originated from deeper, intrascleral tumour cells not visible on fundus imaging. (D–F) After enucleation, the intraocular recurrence appeared as an amelanotic, dome‐shaped tumour (asterisk), and extraocular extension (EXE) was identified posteriorly, measuring 7 mm in largest diameter (arrow).

**FIGURE 2:**
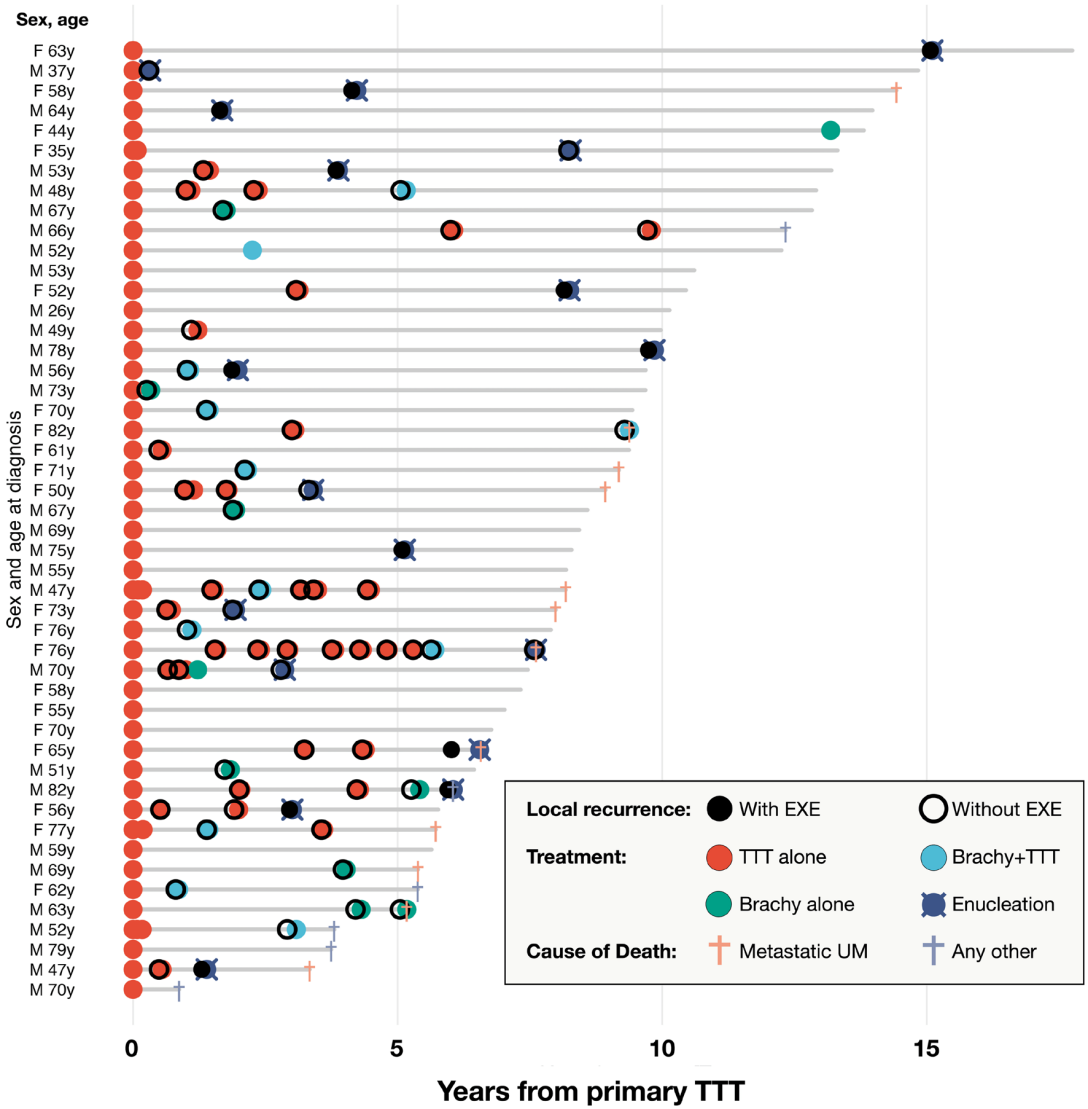
Swimmer plot of treatment timelines and clinical events in patients treated with primary transpupillary thermotherapy (TTT). Each horizontal line represents one patient (*n* = 48) and spans from the date of first TTT to the last recorded follow‐up or death (*x*‐axis, years). Patients are ordered by increasing follow‐up duration. The *y*‐axis shows patient sex and age at diagnosis. Coloured markers indicate subsequent ocular treatments (TTT alone, brachytherapy combined with TTT (brachy+TTT), plaque brachytherapy alone (brachy alone), or enucleation). Local recurrences are shown as open circles (without extraocular extension [EXE]) or filled circles (with EXE). Death is indicated by a dagger (†), colour‐coded by cause (uveal melanoma vs. any other cause). In two cases (F 44y and M 52y), additional treatment was given in the absence of a confirmed local recurrence because of persistent pigmentation within the treated bed that raised concern despite no documented growth; these events are therefore shown without an accompanying recurrence marker.

Among the 48 patients who underwent primary TTT alone, 41 were treated under general anaesthesia and 7 were treated awake at the slit lamp using a TTT module attached to the biomicroscope. Of the 36 patients who developed a local recurrence after primary TTT, 12 did so with EXE, including one case that occurred more than 15 years after the initial treatment. Five patients were biopsied before first treatment. In addition, 18 patients later underwent secondary enucleation for a variety of clinical reasons, including repeated local recurrences with diminishing perceived efficacy of further TTT, peri‐ or circumpapillary extension precluding adequate plaque margins, or extensive recurrences not amenable to safe plaque placement. These procedures provided histopathologic confirmation of the diagnosis in 23 cases. All 12 recurrences with extraocular extension were confirmed histopathologically (Figure 1). The patient who experienced nine separate local recurrences initially had a small tumour measuring 4 mm in LBD and 2.5 mm in thickness and ultimately died with stage IV disease (Figure 2).

Characteristics of the three treatment cohorts—TTT alone, brachy+TTT, and brachy alone—after matching on age at diagnosis, AJCC T‐category, and tumour distance to the optic disc are presented in Table 1. TTT treatment characteristics for patients receiving primary TTT are summarized in Table 2. Tests of proportional subdistribution hazards based on treatment–log(time) interaction terms showed no evidence of non‐proportionality for local recurrence (*p* = 0.47) or local recurrence with EXE (*p* = 0.98), whereas evidence of non‐proportionality was observed for uveal melanoma–related mortality (*p* < 0.001) and death from other causes (*p* < 0.001). Linearity of AJCC stage in the Fine–Gray models was supported for both outcomes (first local recurrence, *p* = 0.25; death from metastatic uveal melanoma, *p* = 0.16).

**TABLE 2:** TTT settings and treatment characteristics for the included patients.

Typical spot size, mm	2.0 or 3.0
Typical exposure time per spot, seconds	30–90 (titrated to faint grey‐white discoloration)
Typical irradiance, W/cm^2^	8–20
Typical treatment margin	1–2 mm beyond visible tumour margins
Mean laser power, mW (median, range, SD)	569 (560, 350–1200, 156)
Mean treatment duration per session, minutes (median, range, SD)	11.4 (10.0, 0.8–22.5, 5.5)
Mean number of treatment sessions per patient, *n* (median, range, SD)	
When used as primary treatment	1.3 (1, 1–3, 0.6)
When used for treating a local recurrence	2.4 (2, 1–7, 1.7)
Total, including patients with multiple local recurrences	2.0 (1, 1–9, 1.6)

Abbreviations: SD, Standard deviation; TTT, Transpupillary thermotherapy.

### 3.2 Survival Analyses

#### 3.2.1 Local Recurrence

**FIGURE 3:**
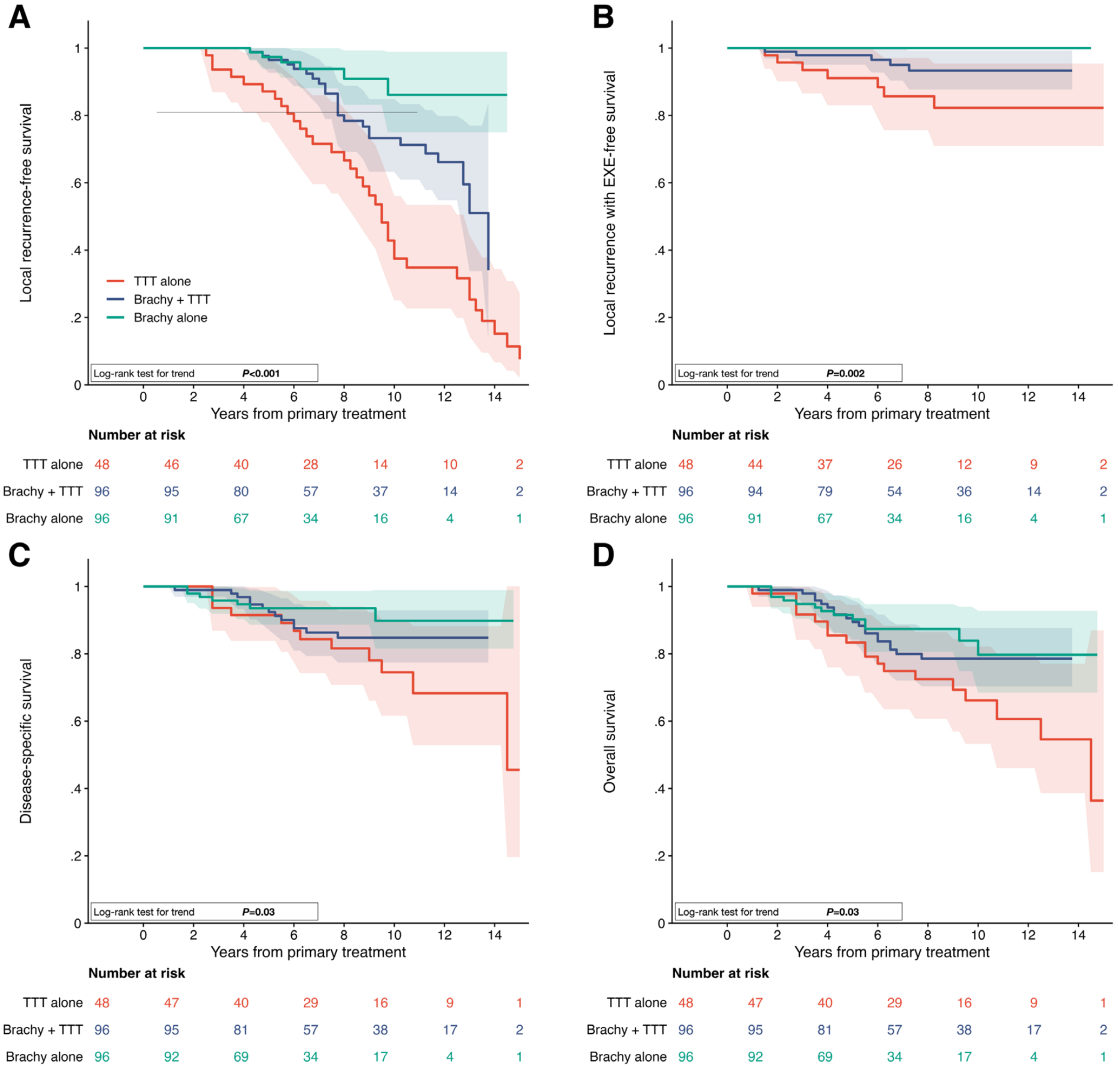
Kaplan–Meier analyses by primary treatment. Kaplan–Meier curves comparing (A) local recurrence‐free survival, (B) local recurrence with extraocular extension (EXE)‐free survival, (C) disease‐specific survival (death from uveal melanoma as the event), and (D) overall survival (death from any cause as the event) across the three primary treatment groups: Transpupillary thermotherapy (TTT) alone, plaque brachytherapy combined with TTT (brachy+TTT), and brachytherapy alone (brachy alone). Time is shown in years from primary treatment. Shaded areas indicate 95% confidence intervals.

Kaplan–Meier local recurrence‐free survival differed between the three treatment groups, with the lowest estimates after TTT alone and the highest after brachy alone. At 15 years, local recurrence‐free survival was 8% (95% CI 0–17) after TTT alone, 34% (95% CI 3–64) after brachy+TTT, and 86% (95% CI 74–98) after brachy alone (log‐rank test for trend *p* < 0.001). Pairwise log‐rank tests showed lower local recurrence‐free survival after TTT alone compared with brachy+TTT (*p* < 0.001) and with brachy alone (*p* < 0.001), and a smaller but still statistically significant difference between brachy+TTT and brachy alone (*p* = 0.03, Figure 3A).

#### 3.2.2 Local Recurrence With Extraocular Extension

When the endpoint was restricted to local recurrence with extraocular extension, the separation between groups persisted but was less pronounced. At 15 years, the local recurrence with EXE‐free survival was 82% (95% CI 70–94) for TTT alone, 93% (95% CI 88–99) for brachy+TTT, and 100%, respectively (log‐rank test for trend *p* = 0.002). Pairwise tests showed lower freedom from local recurrence with EXE after TTT alone compared with brachy alone (*p* < 0.001). Freedom from local recurrence with EXE also tended to be lower after TTT alone than after brachy+TTT, although this comparison did not reach the threshold for statistical significance (*p* = 0.06). Brachy+TTT, in turn, showed lower freedom from local recurrence with EXE than brachy alone (*p* = 0.04, Figure 3B).

#### 3.2.3 Disease‐Specific and Overall Survival

Fifteen years after diagnosis, Kaplan–Meier disease‐specific survival was 46% (95% CI 7–83) after TTT alone, 85% (95% CI 77–92) after brachy+TTT, and 90% (95% CI 81–98) after brachy alone (log‐rank test for trend *p* = 0.03). Pairwise comparisons gave *p* = 0.16 for TTT alone versus brachy+TTT, *p* = 0.04 for TTT alone versus brachy alone, and *p* = 0.31 for brachy+TTT versus brachy alone (Figure 3C).

Overall survival followed a similar pattern, with the lowest estimates after TTT alone. Fifteen‐year overall survival was 36% (95% CI 5–68) after TTT alone, 79% (95% CI 70–87) after brachy+TTT, and 80% (95% CI 68–92) after brachy alone (log‐rank test for trend *p* = 0.03). Pairwise comparisons gave *p* = 0.06 for TTT alone versus brachy+TTT, *p* = 0.03 for TTT alone versus brachy alone, and *p* = 0.55 for brachy+TTT versus brachy alone (Figure 3D).

#### 3.2.4 Competing Risks Analyses

In univariate competing risks regression analyses of the matched cohort, primary TTT alone versus any other primary treatment (i.e., brachy+TTT or brachy alone) was associated with a higher sHR for local recurrence (sHR 3.6, *p* < 0.001) and for death from metastatic uveal melanoma (sHR 2.0, *p* = 0.05, Table 3). In multivariate competing risks regression analyses including all 850 patients and adjusting for AJCC stage, TTT remained associated with an increased sHR of local tumour recurrence (sHR 6.7, *p* < 0.001) and death from metastatic uveal melanoma (sHR 3.9, *p* < 0.001, Table 4).

**TABLE 3:** Multivariate competing risks regression analyses, matched cohorts.

Outcome: local tumour recurrence	Exp (β_j_)	SE	95% CI	*p*
TTT^a^	3.6	0.3	2.1 to 6.1	< 0.001

Outcome: Death from metastatic uveal melanoma	Exp (β_j_)	SE	95% CI	*p*
TTT^a^	2.0	0.4	1.0 to 4.1	0.05

Abbreviation: Exp(β_j_), subdistribution hazard ratio.

^a^ Patients treated with transpupillary thermotherapy (TTT) only as primary treatment (*n* = 48) versus those treated with brachytherapy combined with TTT, or brachytherapy alone (*n* = 192). For the local recurrence model, competing events were enucleation and death from any cause. For the death from metastatic uveal melanoma model, the competing event was death from any other cause.

**TABLE 4:** Multivariate competing risks regression analyses, all patients (*n* = 850).

Outcome: local tumour recurrence	Exp (β_j_)	SE	95% CI	*P*
TTT^a^	6.7	0.3	3.9 to 11.5	< 0.001
AJCC stage^b^	0.6	0.2	0.4 to 0.9	0.01

Outcome: Death from metastatic uveal melanoma	Exp (β_j_)	SE	95% CI	*P*
TTT^a^	3.9	0.3	2.1 to 7.5	< 0.001
AJCC stage^b^	2.0	0.1	1.7 to 2.3	< 0.001

Abbreviations: AJCC, American Joint Committee on Cancer, 8th Edition; exp (β_j_), subdistribution hazard ratio.

^a^ Patients treated with TTT only as primary treatment (*n* = 48) versus those treated with brachytherapy combined with TTT, or brachytherapy alone (*n* = 802).

^b^ Per one‐step increase in AJCC stage (I to IIA, IIA to IIB, IIB to IIIA, etc.). For the local recurrence model, competing events were enucleation and death from any cause. For the death from metastatic uveal melanoma model, the competing event was death from any other cause.

#### 3.2.5 Association Between Number of TTT Sessions and Local Recurrence With EXE

Among the 163 patients who developed at least one local recurrence, EXE was observed only in eyes that had received TTT at some point. Specifically, none of the 88 patients who had a recurrence but had never received TTT developed EXE, whereas 6 of 29 patients (21%) who had received one TTT session and 11 of 46 (24%) who had received two or more TTT sessions eventually developed EXE. When dichotomized as any TTT exposure (≥ 1 session) versus none, the risk of ever developing EXE was 23% (17/75, 95% CI 14%–34%) among TTT‐exposed eyes and 0% (0/88, 95% CI 0%–4%) among eyes never exposed to TTT (Fisher's exact *p* < 0.001).

To separate patient‐level from episode‐level effects, we also analysed all 225 individual recurrence episodes. EXE did not occur in any of the 101 recurrences in eyes without prior TTT exposure. Among recurrences that occurred after prior TTT, EXE was observed in 6 of 65 (9%) after one prior TTT session and 11 of 59 (19%) after two or more prior sessions. Dichotomizing prior exposure as 0 versus ≥ 1 session, EXE occurred in 17 of 124 recurrences (14%) after any prior TTT and in 0 of 101 recurrences (0%) without prior TTT (Fisher's exact test, *p* < 0.001, Supplementary Table 2).

Because EXE did not occur in the reference groups (no TTT exposure), odds ratios are not finite (complete separation) and should not be interpreted as effect‐size estimates. We therefore emphasize the observed absolute risks and the exact test results indicating a strong association between prior TTT exposure and subsequent EXE in this dataset.

#### 3.2.6 Hazard of Another Local Recurrence After Recurrence Treatment

As stated, 163 patients were treated for at least one local recurrence (defined as a second or later surgery), of whom 90 underwent secondary enucleation and 73 patients contributed 131 recurrence‐treatment episodes with eye preserving modalities with TTT alone (*n* = 90), brachy+TTT (*n* = 15), or brachytherapy alone (*n* = 26).

Another local recurrence occurred after 51 of 90 episodes (57%) treated with TTT, 3 of 15 episodes (20%) treated with brachytherapy combined with TTT, and 4 of 26 episodes (15%) treated with brachytherapy alone. Using Fine–Gray competing risks regression, with the date of each recurrence‐treatment episode as time zero, another local recurrence as the event of interest, and secondary enucleation or death from any cause as competing events, treatment with other than TTT was associated with a lower sHR of another local recurrence compared with TTT alone (sHR 0.5, 95% CI 0.2–1.0, *p* = 0.05, Table 5).

**TABLE 5:** Competing risks regression analysis of the risk of another local recurrence after treatment of a previous local recurrence.

Outcome	Treatment	Exp (β_j_)	SE	95% CI	*p*
Another local recurrence after recurrence treatment	Other than TTT^a^	0.5	0.3	0.2 to 1.0	0.05

*Note:* Competing events are secondary enucleation or death from any cause.

Abbreviations: exp(β_j_), subdistribution hazard ratio; TTT, Transpupillary thermotherapy.

^a^ Brachytherapy combined with TTT or brachytherapy alone, with TTT alone as reference category.

This association remained directionally similar when accounting for within‐patient clustering using a patient‐level cluster bootstrap, although confidence intervals widened and crossed unity (bootstrap 95% CI 0.2–1.1). Overall, recurrence treatments performed with TTT alone were followed by a higher cumulative incidence of additional local recurrences over time than treatments involving brachytherapy, although statistical uncertainty remained.

#### 3.2.7 Mediation by Local Recurrence

To assess whether the association between TTT and mortality was mediated by local recurrence, we fitted time‐dependent Cox regressions of disease‐specific and overall survival on treatment, AJCC stage, and local recurrence status as a time‐varying covariate (Supplementary Table 3). Adding the time‐varying local recurrence term attenuated the hazard ratio for primary TTT versus brachy alone from 3.34 to 1.74 for disease‐specific survival and from 2.95 to 1.70 for overall survival. Local recurrence itself was independently associated with both disease‐specific (HR 2.85, 95% CI 1.32–6.15, *p* = 0.008) and overall mortality (HR 2.45, 95% CI 1.32–4.55, *p* = 0.005).

## 4 Discussion

In this single‐centre retrospective cohort, TTT use was associated with higher rates of local recurrence, recurrences with EXE, and lower Kaplan–Meier survival estimates compared with plaque brachytherapy. Despite the small average tumour size at diagnosis in the TTT alone group (mean LBD 6.7 mm and thickness 1.8 mm), long‐term disease‐specific survival was poor, with Kaplan–Meier estimates of 75% at 10 years and 46% at 15 years. These outcomes contrast sharply with the survival observed after brachytherapy, either alone or combined with TTT, which were more consistent with previously reported long‐term outcomes for small choroidal melanomas [27, 28, 29].

Importantly, at our institution, TTT was sometimes selected as primary treatment for lesions suspected to represent atypical choroidal nevi rather than unequivocal melanomas. This diagnostic uncertainty should, if anything, have biased survival in a favourable direction, as such lesions would be expected to include a proportion of tumours with inherently low malignant potential. The observation of poor long‐term survival despite this selection therefore strengthens the inference that TTT was associated with inadequate tumour control. These findings challenge the notion that TTT represents a sufficiently effective or oncologically safe primary treatment modality for choroidal melanoma, even when applied to very small or diagnostically ambiguous lesions [3, 8, 30, 31].

Patients treated with TTT in combination with brachytherapy also experienced worse local recurrence‐free survival and freedom from recurrence with extraocular extension than patients treated with brachytherapy alone. A plausible explanation is that reliance on TTT for tumour control near the optic disc or fovea may have led to reduced brachytherapy safety margins or eccentric plaque placement, undertaken with the intention of sparing critical structures. Rather than compensating for the geometric and dosimetric limitations of plaque brachytherapy in these regions, adjunctive TTT appears to have contributed to under‐treatment of the tumour margins. This is consistent with the observation by Espensen and colleagues that local tumour control after ruthenium‐106 brachytherapy correlates with the minimum tumour dose rather than the prescribed apical dose, since the eccentric plaque placements used in our brachy+TTT cases would be expected to produce lower minimum doses at the posterior tumour margin than centered placements [32].

The 10‐year one minus local recurrence‐free survival of 62% observed in our cohort can be compared with the 42% reported by Shields and colleagues in the United States and the 56% reported by Spire and colleagues in France, although the latter was not based on time‐to‐event analysis [7, 9]. Others have concluded that local recurrence rates after primary TTT, including local recurrences with EXE, are probably too high to be readily acceptable in the field of oncology, including Singh and colleagues in 2008 [33]. Nonetheless, primary TTT has remained in use [34, 35]. Our data extend the previous observations by demonstrating that local recurrences continue to occur at later time points, resulting in an overall local recurrence burden that is clearly worrying. Previous reports suggest that tumour cells that have invaded the sclera may escape destruction by TTT [9]. In turn, scleral invasion has been observed on histopathologic examination in more than 55% to 80% of eyes with small melanomas and may represent a starting point for later recurrences [36, 37]. By contrast, such scleral extensions would be exposed to very high radiation doses during plaque brachytherapy, which may reduce the relative risk of local recurrence compared with TTT. Detailed data on recurrence patterns are not available for this cohort, but clinical experience from these cases indicates that most recurrences after any brachytherapy‐based treatment were marginal, whereas after primary TTT alone a greater proportion were central, consistent with under treatment of the tumour apex where infrared absorption is attenuated by the accumulated depth of tumour tissue.

Despite the large number of reports on ocular outcomes after TTT for choroidal melanoma, the previous literature is surprisingly sparse with respect to long‐term patient survival. Some authors have reported only the number of deceased patients without formal survival analyses, whereas others have focused primarily on local recurrence rates or ocular complications [4, 7, 8, 30]. Spire and colleagues reported outcomes for 18 patients treated with primary TTT for melanomas with a mean largest basal diameter of 6.7 mm and a thickness of 3.2 mm, including 10 local recurrences during a mean follow‐up of 25 months [9]. Their published Kaplan–Meier curve is labelled as metastasis‐free survival, but the number of events and censors matches their reported recurrences, and the curve is most consistently read as local recurrence‐free survival. Long‐term survival data comparable to ours are therefore not available from that series.

The average number of TTT sessions when used as primary treatment was 1.4, which is in line with most previous studies [9, 38, 39, 40]. It is conceivable that more frequent retreatment or stricter patient selection could have reduced recurrence rates. Shields and colleagues reduced their 10‐year one minus local recurrence‐free survival from 42% to 15% with more careful patient selection and treatment of lesions with fewer risk factors for growth [7]. There is therefore a chance that our high rates of local recurrences, and perhaps also mortality, could be reduced with alternative clinical strategies. However, given that a higher number of TTT sessions were associated with higher odds of EXE, the magnitude of the observed differences in local control and the associated increase in metastatic mortality, we have now discontinued the use of TTT for this indication in our clinical practice as a precautionary measure. Our message to international colleagues is not to immediately abandon TTT based on single‐institution data alone, but rather to carefully evaluate their own long‐term outcomes, including patient survival, and to take appropriate measures based on such evaluations.

An important implication of this study is that local recurrence should not be interpreted solely as a marker of intrinsically aggressive tumour biology. Instead, the data support the view that local recurrence can reflect inadequate treatment, which in turn may allow continued tumour growth and additional metastatic seeding. This interpretation is consistent with previous observations that insufficient plaque coverage or suboptimal plaque positioning is associated with increased local recurrence and higher metastatic mortality. In posterior uveal melanoma, use of smaller plaques without verification of plaque position has been linked to higher recurrence rates, with the hazard increasing as the difference between plaque and tumour diameter decreases [15]. Several lines of evidence support the association between continued tumour growth and worse prognosis [41, 42]. This concept is further supported by a recent international collaboration showing that larger primary tumours are associated with higher metastatic burden at detection and shorter survival after the diagnosis of metastatic disease [43]. Together, these findings suggest that effective local tumour control may influence not only the incidence but also the biology and clinical course of metastatic disease. At the same time, intrinsic tumour biology and treatment‐related factors are not mutually exclusive, and both are likely to contribute to the observed association between local recurrence and metastatic outcome.

### 4.1 Strengths and Limitations

This study has several limitations. Because this was a single‐institution study, the findings may reflect local practice patterns and technical factors, and therefore might not be generalizable to other locations. Diagnosis, treatment, and follow‐up were performed by a relatively small group of ophthalmologists using a limited range of equipment, and differences in routines, technique, and device performance across centres could lead to different outcomes. Although matching and multivariable adjustment were used to address measured confounders, the retrospective non‐randomized design limits causal inference. We matched patients based on age, tumour size, and tumour distance to the optic disc, but genetic and cytogenetic prognosticators were not available. Therefore, residual confounding by tumour biology cannot be entirely excluded. However, such confounding would need to operate counter to established knowledge in uveal melanoma, in which aggressive biological features are positively associated with larger tumour size [44].

Second, the number of patients treated with TTT alone was limited, reducing statistical power, particularly for subgroup analyses. Although follow‐up was long for some patients, the number of individuals remaining at risk at later time points was small, resulting in wide confidence intervals.

We did not apply formal correction for multiple comparisons. All hypotheses were defined a priori and addressed distinct, clinically motivated questions rather than exploratory analyses. In this context, strict multiplicity adjustment would increase the risk of type II error and could obscure potentially meaningful associations in an already limited dataset. Nevertheless, applying conservative corrections would likely render several borderline findings non‐significant, underscoring the need for cautious interpretation.

However, the study also has important strengths. These include a well‐defined institutional cohort, long follow‐up, comprehensive capture of local recurrence events, and histopathologic confirmation of all cases with extraocular extension. The main findings were consistent across unmatched and matched analyses, as well as across Kaplan–Meier and competing risks approaches. Notably, the adverse associations observed for TTT were not confined to local tumour control but extended to metastatic mortality, underscoring the clinical relevance of the findings.

Moreover, patients treated with modalities other than TTT alone generally had larger tumours and more advanced disease, even after matching. Any residual imbalances in prognostic factors would therefore be expected to bias outcomes in favour of the TTT alone group rather than against it. This further strengthens the interpretation that the observed differences are unlikely to be explained solely by baseline risk differences between treatment cohorts.

### 4.2 Conclusions

In this single‐centre cohort, TTT use, alone or as an adjunct, was associated with higher local recurrence rates than plaque brachytherapy, and recurrence treatments performed with TTT were followed by more subsequent recurrences than treatments involving brachytherapy. TTT exposure was also associated with melanoma‐related mortality, although residual confounding by tumour biology cannot be excluded. These findings support careful outcome auditing and external validation in cohorts with standardized treatment technique, documented follow‐up intensity, and molecular prognosticators. Following internal review, our centre elected to stop using primary TTT for choroidal melanoma.

## Funding

This study was provided to Gustav Stålhammar from St. Erik Eye Hospital, Stockholm, Sweden. The sponsor or funding organization had no role in the design or conduct of this study.

## Conflicts of Interest

The authors declare no conflicts of interest.

## Supporting information


**Table S1:** Characteristics of all patients and tumours.
**Table S2:** Extraocular extension (EXE) by prior TTT exposure.
**Table S3:** Time‐dependent Cox regression of survival.

## Data Availability

The data underlying this study contain sensitive personal information and are protected under Swedish law. Consequently, the data are not publicly available. Anonymised data may be provided upon reasonable request, contingent on approval by the Swedish Ethical Review Authority.
