# Supplementary material for: Long‐Term Local Control and Mortality After Transpupillary Thermotherapy of Small Uveal Melanomas
Source: Clin Exp Ophthalmol. 2026 May 8;54(6):832–44. doi: 10.1111/ceo.70126 (PMC13462379; doi:10.1111/ceo.70126)
Supplement: Supplementary file 1 [file CEO-54-832-s001.pdf]

Supplementary material

Long-term Local Control and Mortality after Transpupillary Thermotherapy of Small Uveal Melanomas

Comparison with Plaque Brachytherapy in a Single Center

Maria Fili[1,2], Malin Ermedahl Conradi[2], Maya Klaff Dahl[2], Shiva Sabazade[2], Manuel Casselholm De Salles[2], Gustav Stålhammar[1,2,3]

[1]Department of Clinical Neuroscience, Division of Eye and Vision, Karolinska Institutet, Stockholm, Sweden

[2]Ocular Oncology Service, St. Erik Eye Hospital, Stockholm, Sweden

[3]St. Erik Ophthalmic Pathology Laboratory, St. Erik Eye Hospital, Stockholm, Sweden

Table of contents

Supplementary Table 1 2

Supplementary Table 2 3

Supplementary Table 3. 4

Supplementary Table 1

Supplementary Table 1. Characteristics of all patients and tumors

	TTT only, *n*=48	Brachy+TTT, *n*=105	Brachy only, *n*=697
**Sex, *n* (%)**			
Female	20 (42)	51 (49)	351 (50)
Male	28 (58)	54 (51)	346 (50)
**Age at diagnosis, mean (SD)**	61.1 (12.7)	64.6 (12.9)	65.2 (13.5)
**Tumor LBD, mean mm (median, range, SD)**	6.7 (6.5, 2.0–14.0, 2.6)	8.3 (8.0, 2.0–20.0, 3.4)	11.4 (11.0, 1.0–20.0, 3.8)
**Tumor thickness, mean mm (median, range, SD)**	1.8 (1.6, 0.5–4.3, 0.8)	3.0 (3.0, 0.3–6.2, 1.1)	6.0 (5.3, 0.5–10.0, 3.2)
**AJCC T-category, *n* (%)**			
T1a	48 (100)	87 (83)	219 (31)
T1b	0 (0)	0 (0)	9 (1)
T1c	0 (0)	0 (0)	1 (0)
T2a	0 (0)	17 (16)	227 (33)
T2b	0 (0)	0 (0)	9 (1)
T2c	0 (0)	0 (0)	1 (0)
T2d	0 (0)	0 (0)	1 (0)
T3a	0 (0)	0 (0)	168 (24)
T3b	0 (0)	0 (0)	22 (3)
T3c	0 (0)	0 (0)	6 (1)
T4a	0 (0)	0 (0)	27 (4)
T4b	0 (0)	1 (1)	7 (1)
**AJCC stage, *n* (%)**			
I	48 (100)	87 (83)	219 (31)
IIA	0 (0)	17 (16)	237 (34)
IIB	0 (0)	0 (0)	177 (25)
IIIA	0 (0)	0 (0)	57 (8)
IIIB	0 (0)	1 (1)	7 (1)
**Median follow-up, years (IQR, min-max)**			
Metastatic death	6.5 (5.3, 2.6-14.4)	4.7 (1.9, 1.1-7.6)	2.9 (2.6, 0.4-10.3)
Other death	4.3 (2.1, 0.9-12.3)	5.0 (2.0, 2.9-6.7)	3.5 (3.8, 0.4-11.0)
Survivors	9.7 (4.7, 5.8-17.8)	10.0 (4.8, 4.2-14.0)	7.6 (4.8, 3.2-16.0)

This table is descriptive and shows the full 850-patient cohort before matching. Almost all comparative analyses in the main text were performed on the matched cohort in Table 1, restricted to T1a and T2a tumours. The only exception is the multivariable Fine–Gray regression in Table 4, which uses the full cohort with adjustment for AJCC stage. AJCC, American Joint Committee on Cancer. Brachy, plaque brachytherapy. IQR, interquartile range. LBD, largest basal tumor diameter. SD, standard deviation. TTT, transpupillary thermotherapy.

Supplementary Table 2

Extraocular extension (EXE) by prior TTT exposure

Level of analysis	Comparison	EXE / total (%)	Exact test
Patient-level (163 patients with ≥1 recurrence)	Any TTT exposure (≥1)	17/75 (23%)	Fisher's exact *P*<0.001
	vs none	vs 0/88 (0%)	
Recurrence-level (225 recurrence episodes)	Any prior TTT (≥1)	17/124 (14%)	Fisher's exact *P*<0.001
	vs none	vs 0/101 (0%)	

Because EXE did not occur in the unexposed groups (no TTT exposure), odds ratios are not finite (complete separation). The table therefore presents absolute risks and Fisher's exact tests.

Supplementary Table 3.

Time-dependent Cox regression of survival

(a) Disease-specific survival

Covariate	Model A: treatment + stage			Model B: adds time-varying local recurrence		
	HR	95% CI	*P*	HR	95% CI	*P*
Treatment: brachy + TTT (vs brachy alone)	1.58	0.67–3.73	0.30	1.24	0.51–3.03	0.64
Treatment: TTT alone (vs brachy alone)	3.34	1.29–8.63	0.013	1.74	0.60–5.02	0.31
AJCC stage (per step)	7.07	2.99–16.74	<0.001	6.27	2.61–15.06	<0.001
Local recurrence (time-varying, 0→1)	—	—	—	2.85	1.32–6.15	0.008

(b) Overall survival

Covariate	Model A: treatment + stage			Model B: adds time-varying local recurrence		
	HR	95% CI	*P*	HR	95% CI	*P*
Treatment: brachy + TTT (vs brachy alone)	1.48	0.75–2.93	0.26	1.22	0.60–2.46	0.58
Treatment: TTT alone (vs brachy alone)	2.95	1.40–6.25	0.005	1.70	0.73–3.96	0.21
AJCC stage (per step)	5.94	2.92–12.11	<0.001	5.33	2.59–10.96	<0.001
Local recurrence (time-varying, 0→1)	—	—	—	2.45	1.32–4.55	0.005

HR, hazard ratio. CI, confidence interval. TTT, transpupillary thermotherapy. UM, uveal melanoma. Models fitted as Cox proportional hazards regressions on a counting-process (tstart, tstop) representation of the matched cohort, with local recurrence as a time-varying covariate.
